# NOTCH blockade combined with radiation therapy and temozolomide prolongs survival of orthotopic glioblastoma

**DOI:** 10.18632/oncotarget.9275

**Published:** 2016-05-10

**Authors:** Sanaz Yahyanejad, Henry King, Venus Sosa Iglesias, Patrick V. Granton, Lydie M.O. Barbeau, Stefan J. van Hoof, Arjan J. Groot, Roger Habets, Jos Prickaerts, Anthony J. Chalmers, Daniëlle B.P. Eekers, Jan Theys, Susan C. Short, Frank Verhaegen, Marc Vooijs

**Affiliations:** ^1^ Department of Radiotherapy (MAASTRO)/GROW, School for Developmental Biology and Oncology, Maastricht University, Maastricht, The Netherlands; ^2^ Radiation Biology and Therapy Group, Leeds Institute of Cancer and Pathology, St James's University Hospital, Leeds, England; ^3^ Department of Oncology, London Health Sciences Center, London, Ontario, Canada; ^4^ Department of Psychiatry and Neuropsychology, Maastricht University, Maastricht, The Netherlands; ^5^ Translational Radiation Biology, Institute of Cancer Sciences, Wolfson Wohl Cancer Research Centre, University of Glasgow, Glasgow, Scotland; ^6^ Department of Radiation Oncology, Maastro Clinic, Maastricht, The Netherlands

**Keywords:** glioblastoma, RO4929097 NOTCH inhibitor, temozolomide, image guidance radiotherapy, glioma stem cells

## Abstract

Glioblastoma multiforme (GBM) is the most common malignant brain tumor in adults. The current standard of care includes surgery followed by radiotherapy (RT) and chemotherapy with temozolomide (TMZ). Treatment often fails due to the radiation resistance and intrinsic or acquired TMZ resistance of a small percentage of cells with stem cell-like behavior (CSC). The NOTCH signaling pathway is expressed and active in human glioblastoma and NOTCH inhibitors attenuate tumor growth *in vivo* in xenograft models. Here we show using an image guided micro-CT and precision radiotherapy platform that a combination of the clinically approved NOTCH/γ-secretase inhibitor (GSI) RO4929097 with standard of care (TMZ + RT) reduces tumor growth and prolongs survival compared to dual combinations. We show that GSI in combination with RT and TMZ attenuates proliferation, decreases 3D spheroid growth and results into a marked reduction in clonogenic survival in primary and established glioma cell lines. We found that the glioma stem cell marker CD133, SOX2 and Nestin were reduced following combination treatments and NOTCH inhibitors albeit in a different manner. These findings indicate that NOTCH inhibition combined with standard of care treatment has an anti-glioma stem cell effect which provides an improved survival benefit for GBM and encourages further translational and clinical studies.

## INTRODUCTION

Glioblastoma multiforme (GBM) is the most common malignant brain tumor in adults. Multimodal treatment of surgery followed by radiotherapy (RT) and chemotherapy using temozolomide (TMZ) extends the two-year median survival rate of patients from 10% with radiotherapy alone to 27% when combined with temozolomide [1]. However, approximately 50% of brain tumors [2–3] as well as the large majority of the recurrent tumors [4–5] are resistant to TMZ. Tumors often respond to radiotherapy, but recurrence is almost inevitable due to the emergence of radiation resistant cells. Treatment failure leads to a high mortality in GBM patients and therefore, there is a great need for novel treatments that improve clinical management and disease outcome.

In GBM, a subpopulation of radiation resistant tumor cells expressing neural stem cell markers such as CD133 with high proliferative and self-renewal capacity have been shown to contribute to tumor recurrence. These cells are often referred to as glioma stem cells [6–7]. Chemotherapeutic drugs including TMZ as well as radiation therapy were shown to predominantly target the CD133-negative population and as such enrich the CD133-positive population [7–8]. Thus, conventional chemoradiotherapy appears to effectively remove the bulk of tumor cells, while leaving many GBM stem cells alive, driving treatment resistance and tumor relapse. Altogether, it appears that eradication of these cells is needed to augment treatment efficacy and outcome.

NOTCH ligands, receptors and target genes are frequently over-expressed in glioma tissues or cell lines [9–11] and the NOTCH pathway is important in maintenance of glioma stem cells [12]. γ-secretase inhibitors (GSI) effectively inhibit the NOTCH pathway in basic and pre-clinical research as well as clinical trials [13–14]. Inhibition of NOTCH signaling in glioma stem cells has been shown to impair the tumorigenic capacity of these cells and enhance their radiation and chemo-sensitivity [15–18]. Importantly, NOTCH inhibition has also been linked to TMZ-resistance through the EGF containing fibulin-like extracellular matrix protein 1 (EFEMP1) [19]. While these results are promising, the therapeutic potential of NOTCH inhibition still has to be demonstrated in models that are more representative of the clinical situation to fully assess their benefit in the context of standard of care treatment.

In this study, we investigated for the first time the efficacy of a clinically approved NOTCH inhibitor (RO4929097) in tumor control when combined with RT-only, TMZ -only and RT + TMZ treatment groups in a 2D and 3D spheroid model *in vitro* as well as in an orthotopic GBM mouse model. Combining GSI with either RT or TMZ significantly reduced the glioma spheroid growth and tumor progression and prolonged survival when compared with single treatments. This effect was most pronounced with the triple combination (GSI + RT + TMZ) and resulted in an increased tumor growth delay when compared with dual treatments. The expression of glioma stem cell marker CD133 was reduced after single or combined treatments with NOTCH inhibitors, whereas the triple combination also decreased SOX2 and Nestin expression. Our results suggest that one of the underlying mechanisms for the enhanced efficacy of NOTCH blockade when combined with chemotherapy and radiation is a reduced clonogenic survival of glioma stem/progenitor cells.

## RESULTS

### NOTCH pathway is active in GBM cells

**Figure 1:**
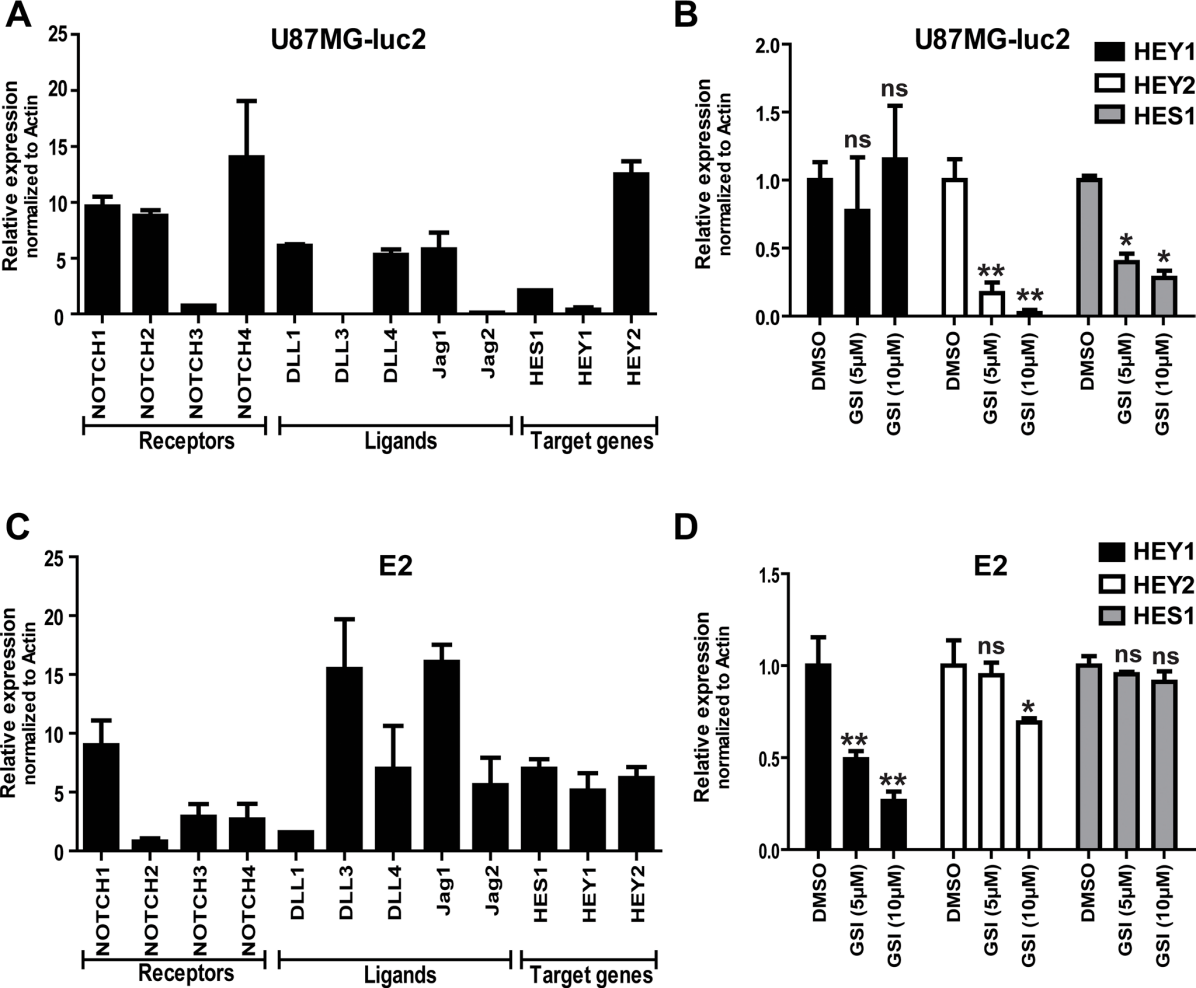
NOTCH signaling in GBM cell lines (**A** and **C**) mRNA expression of the NOTCH receptors, ligands and target genes in GBM cell lines were determined by qRT-PCR in U87MG-Luc2 and E2 cells. (**B** and **D**) mRNA expression of NOTCH target genes (HES1, HEY1 and HEY2) reduced after treatment with different concentrations of clinically available GSI RO4929097 as determined by qRT-PCR. Values were normalized to Actin. Error bars indicate SEM. Asterisk indicates significance (*P < 0.05, **P < 0.01, ns: not significant).

To address if the NOTCH pathway was active in our glioma cell lines, we analyzed the gene expression profile of NOTCH receptors (NOTCH1-4), ligands (DLL 1,3,4 JAG 1,2) and target (HES1, HEY1,2) genes. Differential expression of NOTCH pathway components was confirmed by qPCR in both U87 and primary E2 GBM cells (Figure 1A and 1C). Blocking NOTCH/γ-secretase using 5- or 10 uM of GSI (RO4929097) significantly reduced the expression of the NOTCH target genes HES1 and HEY2 in U87 cells (Figure 1B) and HEY1 and HEY2 in E2 cells (Figure 1D). HEY2 expression in E2 cells was only significantly reduced using 10 uM GSI, while no significant change was observed for HES1 expression (Figure 1D). HEY1 expression in U87 cells was not significantly reduced upon GSI treatment most likely due to the low HEY1 expression in U87 cells (Figure 1A).

### NOTCH inhibition in combination with RT and TMZ attenuates proliferation and clonogenic survival *in vitro*

**Figure 2:**
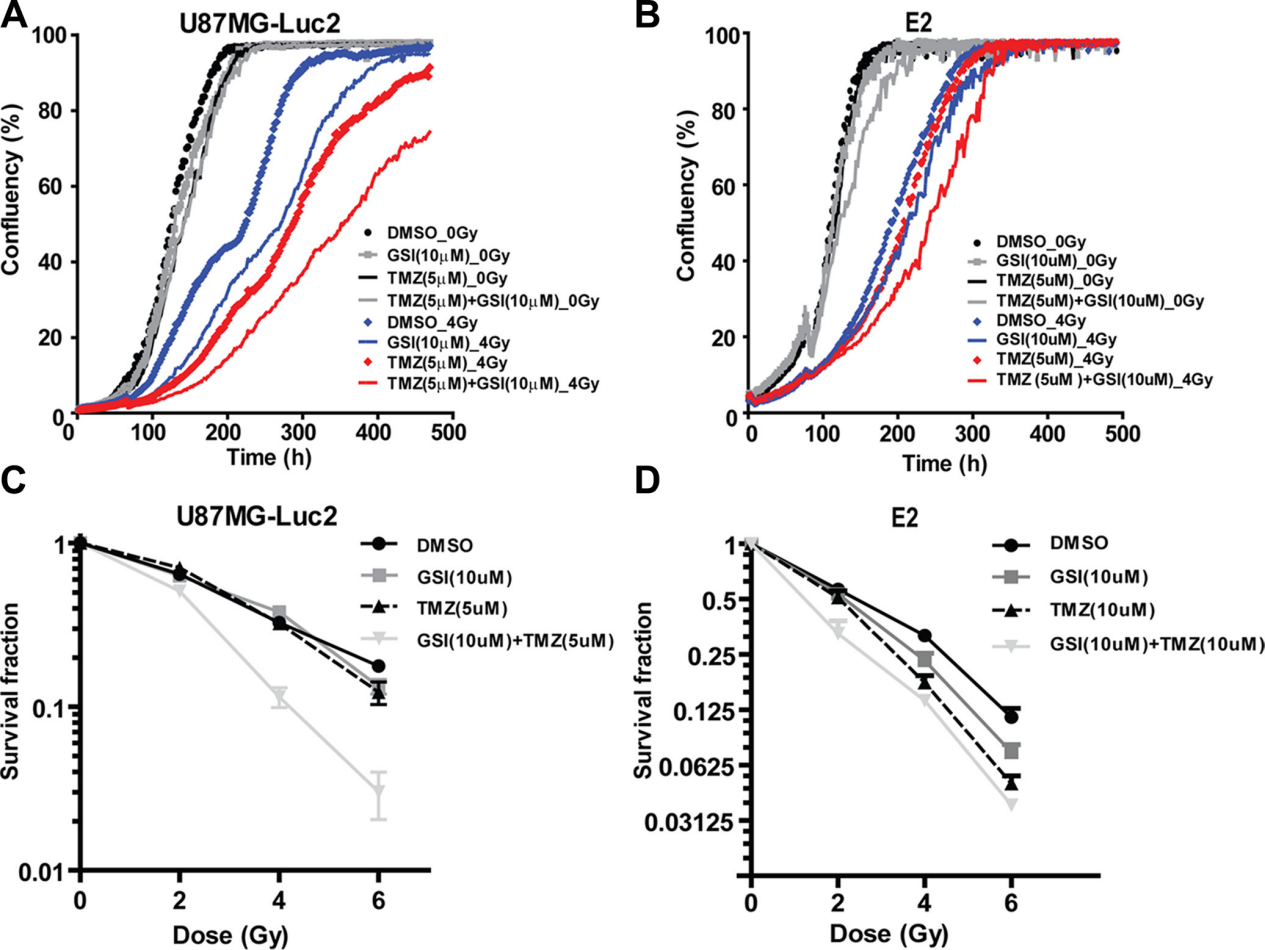
Effect of NOTCH inhibition combined with TMZ and RT on proliferation and clonogenicity *in vitro* (**A**–**B**) Proliferation analysis of U87MG-Luc2 and E2 cells after indicated treatments. (**C**–**D**) Survival fraction of U87MG-Luc2 and E2 cells upon indicated treatments following radiation. Error bars indicate SEM.

Next, we investigated the effect of NOTCH inhibition as mono-therapy or in combination with RT and TMZ on proliferation of U87 and E2 cells in 2D monolayer cultures. In U87 cells, treatment with GSI, TMZ and TMZ + GSI did not affect proliferation significantly compared with the vehicle control (DMSO). Upon RT (4Gy) treatment, GSI-only, TMZ-only and TMZ + GSI significantly reduced proliferation compared to vehicle control (*p* < 0.001, in all cases) (Figure 2A). In E2 primary glioma cells treatment with GSI and TMZ alone did not affect proliferation significantly compared with the vehicle control (DMSO), while TMZ + GSI did (*p* < 0.05). Upon RT (4Gy) treatment, GSI significantly reduced proliferation compared to vehicle control (*P* < 0.01). Similarly, after RT treatment TMZ + GSI significantly reduced proliferation compared to vehicle control, TMZ and GSI treatments (*p* < 0.0001, *p* < 0.001 and *p* < 0.0001, respectively) (Figure 2B).

Next, we examined the effect of combination treatments on clonogenic survival. In U87 cells, upon RT, GSI and TMZ did not affect the clonogenic survival compared with the vehicle control (DMSO), however the survival fraction was markedly reduced in the GSI + TMZ treatment group at 2,4, and 6 Gy and a highly significant synergistic effect was observed (*p* < 0.0001) (Figure 2C). The mean inactivation dose (50% reduction in clonogenicity) for GSI + TMZ was 2.28 Gy and for control + TMZ 3.2 Gy, respectively. In E2 cells, RT, GSI and TMZ reduced the clonogenic survival compared with the vehicle control (DMSO) (*p* = 0.047 and *p* = 0.02, respectively). Clonogenicity was further reduced in GSI + TMZ irradiated cells and this effect was synergistic with 2 Gy irradiation (*p* = 0.0002) (Figure 2D) (Supplementary Figure 1).

**Figure 3:**
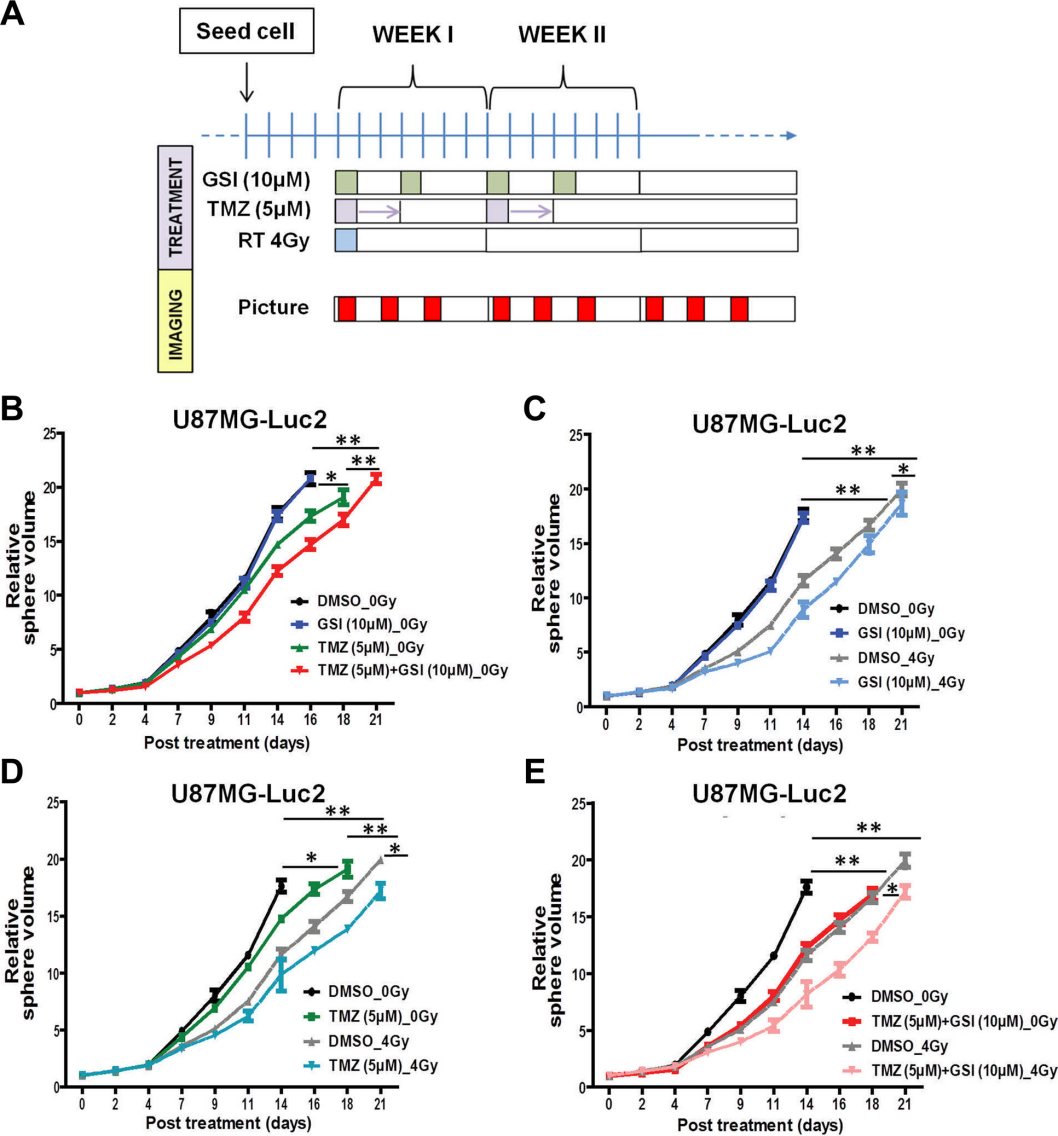
Effect of NOTCH inhibition combined with TMZ and RT on 3D spheroid growth *in vitro* (**A**) Schematic of the 3D spheroid assay treatment schedules. (**B**–**E**) Individual spheroid growth in U87 cells was imaged 3 ×/week and the volume was calculated after indicated treatments till time to reach 20× starting volume. Spheroid growth demonstrates delays upon different treatment combinations compared with DMSO as vehicle control. Error bars indicates SEM. Asterisk indicates significance (*p < 0.05, **p < 0.01 and ***p < 0.001, ns: not significant).

We then investigated the effects of single and combined treatment in a three-dimensional spheroid assay of U87 GBM cells. Four days post-seeding, U87 spheroids were irradiated with a single dose RT (4 Gy) and then treated with TMZ (5 uM) and GSI (10 uM) (Figure 3A).

In the absence of radiation (0 Gy), NOTCH inhibitor alone did not affect the spheroid growth compared with the vehicle control, however when combined with TMZ an enhanced effect was observed (*p* = 0.01) (Figure 3B). RT treatment significantly delayed spheroid growth (*p* = 0.005), which was further enhanced upon treatment with GSI compared to non-RT treated cells (*p* = 0.007) (Figure 3C). Finally, GSI combined with TMZ and single dose RT treatment (GSI + TMZ + RT) resulted in the most pronounced growth delay when compared with either treatment combination including the standard of care, TMZ + RT, (*p* = 0.02) (Figure 3D–3E). Representative images of spheroids in each treatment group at different time points post-treatment are shown (Supplementary Figure 2). For E2 cells, spheroids formed but were not able to grow consistently over time in agarose-coated plates (not shown). Therefore, we performed the 3D spheroid assay in another primary GBM cell line; G7 (Supplementary Figure 3). In the absence of radiation (0Gy), NOTCH inhibitor alone did not affect the spheroid growth compared with the vehicle control, however when combined with TMZ a significant growth delay was observed (*p* = 0.02) (Supplementary Figure 3A). RT treatment also significantly delayed spheroid growth (*p* = 0.01), which was further enhanced upon treatment with GSI compared to control (*p* = 0.03) (Supplementary Figure 3B). Finally, the most pronounced growth delay was observed in spheroids treated with GSI + RT + TMZ (*p* = 0.03) (Supplementary Figure 3C–3D). There was no significant interaction between GSI and RT + TMZ (two-way ANOVA; *P* > 0.05), the effect of the combination therapy in G7 was additive.

### NOTCH inhibition combined with standard of care treatment reduces tumor growth and prolongs survival of intracranial glioblastoma *in vivo*

**Figure 4:**
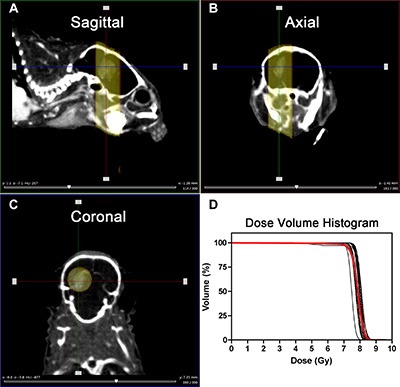
Radiation treatment set-up and the resulting dose volume histograms (**A**–**C**) Visualization of the tumor in the brain from different planes (Sagittal, Axial and coronal) and applied parallel-opposed radiation beams to target the tumor. (**D**) Resulting DVHs of the tumors after 8 Gy irradiation. Red line shows the average of the DVHs.

**Figure 5:**
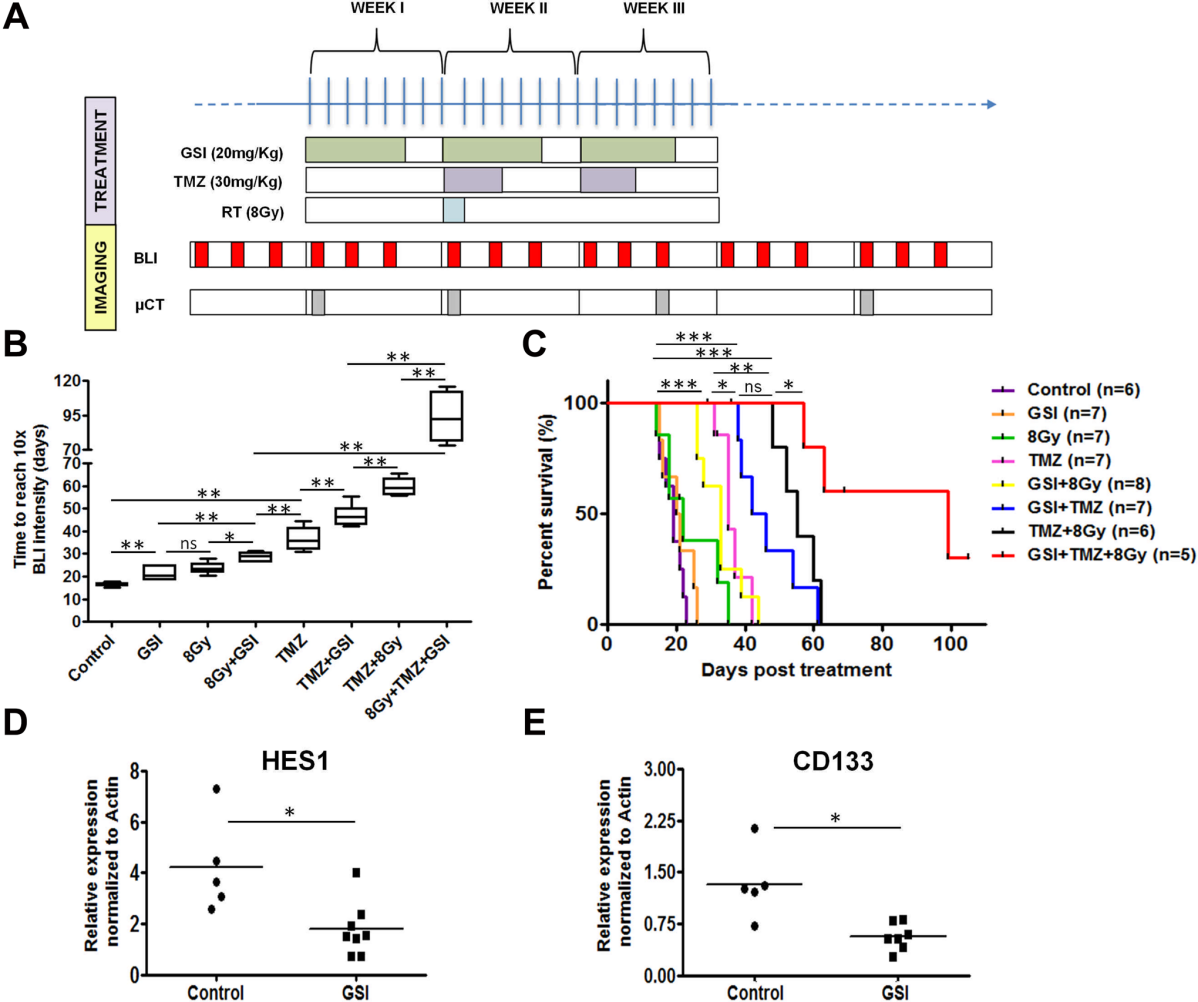
Effect of NOTCH inhibition combined with TMZ and RT in orthotopic U87MG-Luc2 glioblastoma *in vivo* (**A**) Schematic of the treatment schedules *in vivo*. (**B**) Tumor growth delay upon indicated treatments measured by BLI signal intensity for mice in each treatment group and the end point was when the BLI signal intensity reached to 10× intensity of the starting treatment day. (**C**) Kaplan-meier survival curve indicates number of days mice survived post-treatment. End-point was assessed based on the neurological sign and weight loss. (**D**–**E**) mRNA expression of the HES1 and CD133 in tumor samples treated with GSI *vs.* control was determined by qRT-PCR. Values were normalized to Actin. Error bars indicates SEM. Asterisk indicates significance (*p < 0.05, **p < 0.01 and ***p < 0.001, ns: not significant).

Next, we assessed the effects of NOTCH inhibition in combination with TMZ and single dose RT on the survival of mice with U87 intracranial glioblastoma. We previously determined that single dose RT of 8 Gy results in a significant but modest tumor growth delay compared to tumors that did not receive any irradiation [20], thereby providing a window of opportunity where the combined effects of RT and TMZ could be measured. One week post-implantation, mice were imaged with BioLuminescence Imaging (BLI) 3 ×/week. Contrast-enhanced micro-CT was also used to monitor tumor growth periodically as reported previously [20]. Upon confirmation of tumor growth by either BLI or micro-CT, mice were randomized into eight different treatment arms. These mice were treated with GSI/vehicle, TMZ/vehicle as well as radiation (Figure 5A) using a dedicated small animal irradiator and associated treatment planning software (SmART-Plan) to create irradiation plans to deliver a conformal dose of 8 Gy to the tumor with minimal normal tissue exposure. An example of the parallel-opposed radiation treatment in sagittal, axial and coronal planes (Figure 4A–4C) and the dose volume histograms (DVHs) for all irradiated mice is shown (Figure 4D). These DVHs demonstrate that a highly uniform dose distribution across the tumor volume is achieved at the desired prescription dose of 8 Gy [20].

We determined the tumor growth delay using bioluminescence by calculating the time it takes for each tumor to reach 10 × BLI signaling intensity from start of treatment (T10 × SI). A significant growth delay was observed in GSI-, 8 Gy- and TMZ-only treated groups (21.3 ± 2.9 days, 23.7 ± 2.6 days, and 37.1 ± 5.1 days, respectively) compared to the control group (16.4 ± 0.9 days) (*p* < 0.01 for GSI- and 8 Gy- and TMZ-only groups) (Figure 5B). The median survival defined based on the weight loss, neurological signs and abnormal behavior as humane endpoints for the GSI- and 8 Gy- single treatments were not significantly different from the control (20.5 ± 4.5 days for GSI-only, 20 ± 8.4 days for 8 Gy-only, 19 ± 3.0 days for control) (Figure 5C). Addition of GSI to either TMZ or 8Gy irradiation resulted in a significant growth delay (47.1 ± 4.5 days and 28.6 ± 1.9 days, respectively) when compared with 8 Gy- or TMZ-only groups (*p* < 0.01) (Figure 5B). The median survival for these groups was significantly prolonged compared to control (33 ± 6.3 days for GSI + 8 Gy and 44 ± 9.1 days for GSI + TMZ, *p* < 0.001 for both groups) (Figure 5C). Similarly, the standard of care treatment (8 Gy + TMZ) resulted in a significant growth delay (59.6 ± 3.8 days) when compared with 8 Gy- or TMZ-only groups (*p* < 0.01) as well as the TMZ + GSI group (*p* < 0.01). The median survival for this group was significantly prolonged compared to single treatments (55 ± 5.7 days for 8 Gy + TMZ, *p* < 0.001 comparing with 8 Gy- and TMZ-only). The most profound growth delay (93.6 ± 18.4 days for 4 out of 5 mice, *P* < 0.01 compared with 8Gy + TMZ) and increase in median survival (81 ± 22.7 days for 4 out of 5 mice, *p* < 0.001 compared with control and GSI + 8 Gy, *p* < 0.05 compared with GSI + TMZ and 8 Gy + TMZ) was observed in mice treated with triple combination of 8 Gy, TMZ and GSI (Figure 5C). Addition of GSI to TMZ + RT resulted in a highly significant synergistic effect (*P* < 0.0003). One out of 5 mice showed a cure defined as being tumor-free for > 14 weeks post start of treatment. Representative BLI and contrast-enhanced micro-CT images of a mouse in each treatment group at different time points post-treatment are shown (Supplementary Figure 4). We observed significant down regulation of HES1 and CD133 mRNA expression in tumors from GSI treated mice, compared to control treated mice (*p* = 0.01) (Figure 5D–5E). HEY2 mRNA was not altered upon GSI treatment (Supplementary Figure 5).

No dose limiting toxicities were observed for single and double treatments although weight loss < 20% was invariably observed within the first or second week after treatment after which mice recovered (Supplementary Figure 6). Importantly, four out of nine mice receiving triple combination treatments were euthanized due to a significant weight loss prior to reaching endpoint despite very small histologically confirmed tumors.

### Combination treatment (GSI + TMZ + RT) reduces the glioma stem cell population

**Figure 6:**
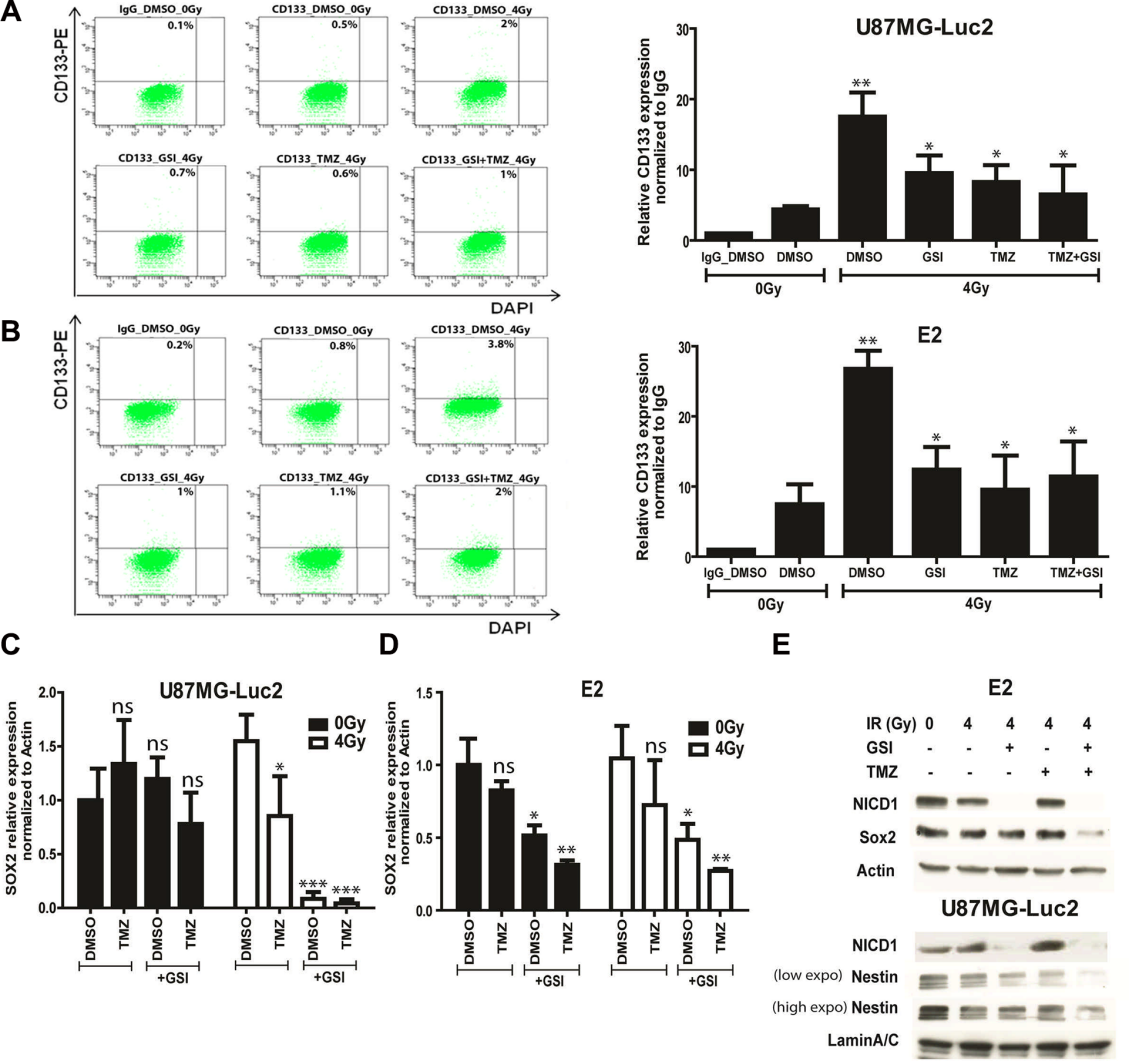
Effect of NOTCH inhibition combined with TMZ and RT on CD133, SOX2 and Nestin expression (**A**–**B**) The expression of CD133 marker upon indicated treatments in U87MG-Luc2 and E2 cells was measured by flow cytometry. Graphs indicate the quantification of the relative CD133 expression normalized to IgG control upon indicated treatments. DAPI was used for the live cell staining. Concentration used in all cases was 10 μM GSI and 10 μM TMZ. (**C**–**D**) mRNA expression of the SOX2 stem cell marker in U87MG-Luc2 and E2 cells upon indicated treatments was measured by qRT-PCR. E) Protein expression of SOX2, Nestin and active form of NOTCH1 (NICD1) in E2 and U87 cells upon indicated treatments was analyzed by Western blotting. Actin and Lamin A/C serve as loading controls. Concentration used in all cases was 10 μM GSI and 10 μM TMZ. Error bars indicate SEM. Asterisk indicates significance (*p < 0.05, **p < 0.01 and ***p < 0.001, ns: not significant).

To investigate the mechanism of increased radio- and chemo-sensitivity upon NOTCH inhibition, we evaluated the expression of the glioma stem cell marker CD133 using flow cytometry 4 days post-treatment in U87 and E2 cells grown under adherent stem cell conditions (Figure 6A–6B). We observed a 4-fold increase in CD133 expression in primary E2 and U87 cells upon single dose 4 Gy RT. Pre-treatment with either GSI or TMZ reduced CD133 expression by 2.1 and 2.9 fold in primary E2 cells as well as 1.8 and 2 fold in U87 cells, respectively (Figure 6A–6B). No significant differences were observed in TMZ + GSI treated cells. Under the same treatment condition, SOX2 mRNA expression was significantly reduced upon GSI + RT and GSI + TMZ + RT treatments in both E2 and U87 (Figure 6C–6D). Further, a strong reduction in SOX2 and Nestin protein expression in E2 and U87 cells, respectively, were only observed in the triple combination (Figure 6E). GSI strongly inhibited NOTCH cleavage in E2 and U87 cells as shown by immunoblotting with Val1744 recognizing the active cleaved form of NOTCH1 (NICD1) (Figure 6E).

## DISCUSSION

Given the lack of a curative treatment for GBM, novel approaches are urgently needed in relevant *in vitro* and preclinical models that are predictive and mimic clinical routine to enable swift translation. Here, we show the therapeutic benefit of the addition of the clinically approved NOTCH/γ secretase inhibitor (RO4929097) combined with the standard of care in GBM: concurrent radiotherapy and chemotherapy with temozolomide. Our results show that in an orthotopic model for glioblastoma, NOTCH inhibition enhances radiotherapy, TMZ chemotherapy as well as a combination of both such that in 1 out of 5 mice tumor cure (defined as being tumor-free for >14 weeks) was achieved and in the remaining cohort a survival benefit of ~62 days and ~26 days was obtained when compared to the untreated mice or the standard of care treatment group (TMZ + RT), respectively.

Previously, we have shown the rationale for using the 3D spheroid system to model clinical efficacy of combination treatments [20]. Consistently, in this study we have shown that *in vitro* screening of NOTCH inhibitors combined with chemo-radiation (GSI + TMZ + RT) using 3D spheroid system is a robust methodology to identify novel synergistic combination therapies for GBM that reflect *in vivo* response. The 3D model used here however still lacks important factors from the tumor micro-environment that are known to contribute to response such as vasculature, fibroblasts or immune cells. Therefore, we used our previously established orthotopic GBM model [21] to address the *in vivo* efficacy of the NOTCH inhibitors combined with chemoradiation. Radiation-induced necrosis of normal brain tissue is a dose-limiting factor in treatment of CNS tumors. Considering this and in contrast to standard devices using a single large beam, we used an image-guided small animal micro-irradiator device that combines micro-CT imaging with conformal irradiation and millimeter-adjustable beams [20]. This was combined with treatment planning software (SmART–Plan) which made radiation treatment planning and delivery highly accurate. Similar to that in clinical practice, we determined dose-volume histograms (DVH) showing that the X-ray beams were highly conformal and localized to the tumor region, minimizing exposure to organs at risk such as the normal brain [20–22].

Based on our findings, growth responses between the *in vitro* spheroid and *in vivo* preclinical models did complement one another; however, there were some discrepancies as well. In accordance with the observation from other studies [16–17], we showed that NOTCH inhibition alone did not affect the spheroid growth in U87 cells but did result in a significant growth delay *in vivo* according to the BLI data. One explanation could be the contribution of NOTCH inhibition in the tumor microenvironment possibly by disrupting tumor angiogenesis [23] or blocking endothelial to tumor cell signaling [24–25], which was not addressed in this study. Our findings are in agreement with previous results showing the enhanced therapeutic effect of combined GSI with TMZ (*in vitro* and *ex vivo*) or GSI with RT (*in vitro* and *in vivo*) compared to each treatment alone [16, 24, 26]. While these findings are important to move NOTCH inhibitors forward as GBM therapeutics, contribution of GSI to current standard of care treatment (TMZ + RT) has never been reported to the best of our knowledge. In this study, for the first time we show that NOTCH inhibition in combination with TMZ + RT significantly enhances the 3D spheroid growth delay *in vitro* and prolongs survival of the mice bearing the orthotopic glioma compared to TMZ + RT. This result is encouraging in developing new combination therapy for GBM; however, the clinical relevance of this study could further be maximized by the use of patient derived xenografts that may better reveal the gene expression profiles of the glioma patients and typical histological characteristics (micro-infiltrative, highly vascularized, palisading necrosis) compared to established glioma cell lines [27]. Recently, integrated genomic profiling of glioblastoma has led to new classifications with prognostic and predictive importance [28–31]. Targeted therapeutics against ‘actionable’ targets has however led to limited clinical efficacy in GBM [32]. This may in part be due to high heterogeneity in GBM and clonal expansion driven by treatment or de novo acquired resistance mechanisms [33]. Therapeutics targeting the survival of tumor initiating cells are more likely to be successful as they target “driver” populations. Identification of these driver populations needs further investigation but our work and that of others highlight the importance of glioma CSCs in growth and treatment response and their dependence on NOTCH signaling.

We observed that NOTCH inhibition influenced the radiation-enhanced CD133 expression, in line with the expected role of NOTCH in the maintenance of glioma stem cells [24]. Importantly, it has been reported that endothelial cells can function as a stem cell niche to promote CD133 + self-renewal in glioblastoma [25] by providing NOTCH ligands that activate NOTCH receptors in CD133 + cells [24, 34]. If NOTCH inhibition also reduced CD133 + sub-populations directly or indirectly via endothelial cell signaling *in vivo* was not addressed in our study and requires further investigation.

Our data showed that temozolomide was equally effective as single NOTCH inhibitor treatment or dual treatment in reducing radiation increase of CD133 + cells, consistent with other reports [35]. This finding could only be partly replicated in our clonogenics assays (not enriched for CD133 +) suggesting that while CD133 + marks clonogenic cells, it does not reflect only glioma stem cells. Others report that CD133^+^ cells are resistant to chemotherapy [8, 36]. However, in some of these studies the viability of CD133^+^ cells was assessed only in short-term culture using extremely high TMZ concentrations (0, 2–2 mM) and culture conditions not suited for the maintenance of the cancer stem cell phenotype [37–38]. In line with our data, TMZ treatment (5–25 uM) on glioma cells grown under stem cell enriched conditions reduced proliferation and survival at very low TMZ concentrations [39] and inhibited the growth of neurosphere in CD133^+^ sub-population and reduced tumorigenicity [35, 40]. While further investigation is needed to reconcile these findings, our data suggest that within the CD133^+^ population a treatment resistant clonogenic population exist sensitive to NOTCH inhibition which support this study and other studies showing that NOTCH inhibitors can sensitize to TMZ [16].

While the exact mechanism for the enhanced efficacy of the triple combination (GSI + RT + TMZ) treatment *in vivo* is not investigated, the marked reduction in clonogenic survival upon triple combination treatment suggests an anti-glioma stem cell effect. Although triple treatment did not further reduce CD133^+^ surface population compared to dual treatments, it reduced SOX2 expression a widely used marker for repopulating cells in context of radiation resistance [41–42] as well as Nestin another stem cell marker in glioma progression [43–44]. This suggests that the treatment resistant population sensitive to NOTCH inhibitors is contained within the CD133, SOX2 and Nestin overlap expression. The phenotypic and functional identity of this population *in vivo* would be a key step forward in understanding glioma treatment resistance [45].

Various clinical trials are underway to investigate whether NOTCH blockade using γ-secretase inhibitors improves cancer treatment [46]. However, one of the major challenges on the way is the untoward side effects associated with NOTCH inhibitors, especially the cytotoxicity in the gastrointestinal tract [47]. To maximize the therapeutic effects and minimize the systemic NOTCH-related side effects, improved dosage regimens have now been reported in phase 1 clinical trials that “spare” the intestine [48]. In our study, using the same orally administrated GSI RO4929097 with similar scheduling (5 days on - 2 days off/for 3 weeks), did not lead to irreversible weight loss or other toxic effects. However, we did observe deaths not due to the tumor burden but rather due to toxicity associated with the prescribed triple therapy regimen by a yet unknown mechanism. We speculate that this could be due to toxicity associated with the three-drug combinations. It is in our estimation that the TMZ component of the treatment was likely the cause of this toxicity, even though we used the lowest reported dosages found in the literature. In the future study, using a lower chemotherapy dose to cause a mild but significant growth delay could be a potential option to prevent toxicity from triple combinations. Furthermore, balancing efficacy and toxicity of γ-secretase inhibitors by optimizing treatment cycles and dosing schemes is paramount for successful future clinical applications. In this regard, a current clinical trial is recruiting patients to investigate the efficacy of RO4929097 in combination with radiation and TMZ (http://ClinicalTrials.gov identifier NCT01119599). Importantly, both our *in vitro* and *in vivo* data indicate that the addition of GSI to either RT or TMZ may be equally effective as standard of care. This could be clinically significant in treatment of recurrent (TMZ resistant) tumors where RT and/or TMZ could be replaced with GSI. Taken together, we believe that our findings are promising for clinical translation to increase survival in GBM patients but that unanticipated toxicities may occur. Therefore, identification of a subset of patients who have active NOTCH signaling could predict the likelihood of increased response to anti-NOTCH therapy. More studies are needed to fully exploit the potent NOTCH inhibitors but also to recognize potential aggravating conditions that may occur.

## MATERIALS AND METHODS

### Cell line culture

U87MG-Luc2 (U87), primary E2 and G7 glioma cell lines were used in this study. E2 and G7 cells were derived from freshly resected human GBM specimens as previously described [49]. U87 cells were cultured in Hyclone MEM/EBSS (Fischer Scientific), 10% fetal calf serum, 1% L-Glutamine (Invitrogen), 1% non-essential amino acids (Invitrogen) and 1% sodium pyruvate (Invitrogen). E2 and G7 cells were cultured in MEMα (Gibco) supplemented with 5% L-glutamine. The U87 cell identity was confirmed using the short tandem repeat (STR) analysis (Identicell, Denmark).

For western blotting and flow cytometry both cell lines were cultured in stem cell enriching conditions in Advanced DMEM F12 medium (Gibco) supplemented with 1% B27 (Invitrogen), 0.5% N2 (Invitrogen), 4 μg/ml heparin, 20 ng/ml fibroblast growth factor (bFGF, Sigma), 20 ng/ml epidermal growth factor (EGF, Sigma) and 1% L-Glutamine. Stem cell enriched cultures were grown on Matrigel (BD Biosciences) coated flasks (1:50 dilution) in serum free media.

### Orthotopic brain tumor implantation and drug treatment

Immunocompromised CD1 nu/nu mice were used in this study. Animal work was performed in accordance with national guidelines. The procedure for tumor implantation has been described in detail previously [21]. After confirmation of tumor establishment, GSI was orally administered with a schedule of five days on and two days off for 3 weeks. One-week post-treatment GSI/vehicle mice were irradiated. Directly after irradiation, mice were treated with TMZ/vehicle with a schedule of 3 days on and 4 days off for 2 weeks. The control group received an intraperitoneal (i.p.) injection of 200 μl saline (TMZ-vehicle), and/or oral administration of 200 μl of 1.0% carboxymethyl cellulose with 0.2% Tween 80 in sterile water (RO4929097-vehicle). The TMZ-only treated group received 30 mg/kg TMZ (Selleckchem) i.p. and oral RO4929097-vehicle, while the RO4929097 treated group received 20 mg/kg RO4929097 orally and TMZ-vehicle i.p. RT-only group received 8 Gy single dose irradiation using the small animal micro-IR (X-RAD 225Cx, Precision X-ray Inc., North Branford, CT). The combined treatment group was administered with the same concentration of TMZ i.p., RT and RO4929097 orally. RO4929097 (2,2-dimethyl-N-((S)-6-oxo-6,7-dihydro-5H-dibenzo (b, d) azeptin-7-yl)-N’-(2, 2, 3, 3, 3,-pentafluoro-propyl)-malonamide) has been indicated as GSI in this study. The RO492909 was stored at 4°C.

### *In vivo* imaging

Bioluminescent imaging (BLI) was performed using the Optix MX2 system (ART Inc., Saint-Laurent, QC). Animals were monitored 3×/week. Additionally, a selection of animals in each group were followed by contrast-enhanced micro-CT imaging using the small animal micro-IR (X-RAD 225Cx, Precision X-ray Inc., North Branford, CT). All the procedures for imaging and analysis have been extensively described previously [21].

### *In vivo* irradiation: (SmART-Plan)

Radiotherapy dose was calculated and prescribed using the dedicated small animal radiotherapy planning system SmART-Plan (v1.3.1, Precision X-Ray, North Branford, CT), which has been previously validated [22]. Target delineation, treatment planning and irradiation was performed on anesthetized mice based on a contrast-enhanced micro-CT images that were shown to successfully visualize tumor boundaries as described previously [20, 21], and was completed while the animal remained anesthetized on the treatment bed. Within SmART-Plan the tumor was contoured and two 5-mm parallel-opposed beams were placed at the center of the tumor. This beam size ensured complete coverage plus a small margin for set-up uncertainties but still avoided irradiating most of normal brain tissue. Based on earlier work, a homogeneous dose of 8 Gy was prescribed to the target with each beam contributing an equal amount of dose. The source-to-axis distance was 30 cm. Radiation was delivered using the small animal irradiator at 225kVp, 12 mA (filtration of 0.3 mm of copper) which provides a dose rate of approximately 3 Gy/minute.

### 3D spheroid assay

96-well plates were coated with 50 μl of autoclaved 1.5% agarose in serum free medium [50]. 2800 U87 and 5000 G7 cells in 200 μl complete medium were seeded on the agarose coated plates per well. One spheroid per well was formed within 4 days after seeding. For each condition, at least 12 spheroids were tested. 4 days post-seeding first phase-contrast pictures were taken from all the spheroids individually and then the sphere volume was analyzed using an in-housed script using Matlab program (Mathworks, 2009b, CT) and available for download [51]. Directly after image acquisition, treatment regimens were administered. GSI (RO4929097) and TMZ were administered immediately after irradiation in RT treated groups. The control group received only DMSO. Final concentration for GSI-only and TMZ-only treated group was 10 μM and 5 μM, respectively for U87 cells and 20 μM and 5 μM, respectively for G7 cells. The RT-only group was irradiated with a single dose of 4 Gy. All the combination groups were treated with the same doses. So as not to disrupt the sphere structure, in all treatment groups 100 μl of medium was discarded very carefully and 100 μl of the 2× treatment pre-mixtures were added. The drugs were washed out by refreshing the medium 3×/week. TMZ was washed out after three days incubation in the first week and the same treatment procedure was repeated in the second week, while GSI was refreshed twice weekly for two weeks. TMZ was diluted in DMSO and stored in 100 mM stock at −80°. GSI was diluted in DMSO and stored in 10 mM stock at −20°. Sphere volumes were measured 3 times a week and experiments were terminated when treated spheres reached 20× treatment volume.

### Proliferation

500 cells/well (U87) or 2000 cells/well (E2) were seeded in 96 well plates and were treated 24 h post-seeding with 5 μM TMZ and/or 10 uM GSI (RO4929097) and/or single dose of 4 Gy RT (Philips X-ray tube; 225 kV; 10 mA). The three treatments were tested as single, double and triple combinations with 6 replicates per condition. 72 h post-treatment TMZ was washed out and GSI was refreshed. Cell confluency was monitored every 2 h using the phase-contrast mode of the IncuCyte^™^ FLR (2011A) live-imager. TMZ was diluted in DMSO and stored in 100 mM stock at −80°. GSI was diluted in DMSO and stored in 10 mM stock at −20°.

### Quantitative real time PCR (qPCR)

RNA extraction was performed using the NucleoSpin RNA II kit (Bioke). cDNA was prepared using the iScript cDNA Synthesis kit (BioRad). Reactions were carried out in a 10 μl volume using sensiMix SYBR low-ROX kit (GC Biotech) with the ABI Prism 7500 Sequence Detection System. Values for each gene were normalized to expression levels of Actin RNA. Primer sequences have been described previously [52].

### Clonogenic survival assay

Cells were seeded in 6-cm dishes to be 70% confluent at the time of irradiation. Cells were irradiated with 2 Gy, 4 Gy or 6 Gy and non-treated (Philips X-ray tube; MU15F/225, 225 kV; 10 mA). Directly after irradiation, cells were trypsinized and seeded at densities of 250 or 1000 cells per well for E2 and 500 cells per well for U87 in triplicate in 6-well plates and allowed to adhere for 4 h in complete medium. Then, the medium was replaced with treated medium: DMSO in control group, 10 μM TMZ for E2 cells and 5 μM TMZ for U87 cells for 48 h in TMZ-only treated group and 10 μM RO4929097 in GSI-only treated group. The combination groups were also treated with the same concentrations. RO4929097 was refreshed every 5 days during the experiment. Colonies were counted manually after 2 weeks. The minimum number of cells per colony was 30.

### Flow cytometry

Cells were first irradiated with 4 Gy and then treated with 10 μM RO4929097 and 10 μM TMZ as well as their combination. Cultures were disaggregated with accutase 4 days post-treatment, cells were washed with PBS once and incubated with CD133/2-PE (1:200; Miltenyi Biotech) or isotype control antibody on ice for 30 min. Then cells were washed with PBS 2×, resuspended in 200 μl PBS and fixed in 1% paraformaldehyde. Just before measurements of each FACS tube DAPI (final concentration 3 μM) was resuspended in each tube. FACS analysis was carried out on FACS Calibur machine (BD Biosciences).

### Western blotting

Cells were first irradiated with 4 Gy and then treated with 10 μM RO4929097, 10 μM TMZ as well as their combination. 4 days post-treatment total cell lysate was prepared using SDS lysis buffer. Lysates were blotted onto a PVDF-membrane. Membranes were probed overnight at 4°C with primary antibodies and bound antibodies were visualized using HRP-linked secondary antibodies (Cell-Signaling) and ECL Luminescence (Pierce Biotechnology). Anti-SOX2 (ab75485, Abcam), anti-Nestin (ab6320, Abcam), anti-beta-tubulin III (T3952, Sigma-Aldrich), and anti-Lamin A/C (Sigma-Aldrich) were used at 1:1000 dilution.

### Statistical analysis

Statistical analysis was performed using GraphPad Prism Software (v5.02, San Diego, CA). For all measured quantities mean ± SEM are reported except where stated. For survival data median ± SD is reported. For tumor growth delay mean ± SD is reported. Mann-Whitney or ANOVA tests were used to analyze differences in growth delay and in clonogenic survival. We used a two-way ANOVA to test the interaction (synergism) between GSI and TMZ + RT. The log-rank (Mantel-Cox) test was used to compare the survival curves. A *p*-value smaller than 0.05 was considered statistically significant.

## SUPPLEMENTARY FIGURES


