# Supplementary material for: NOTCH blockade combined with radiation therapy and temozolomide prolongs survival of orthotopic glioblastoma
Source: Oncotarget. 2016 May 10;7(27):41251–64. doi: 10.18632/oncotarget.9275 (PMC5173056; doi:10.18632/oncotarget.9275)
Supplement: Supplementary file 1 [file oncotarget-07-41251-s001.pdf]

NOTCH blockade combined with radiation therapy and temozolomide prolongs survival of orthotopic glioblastoma

Supplementary Materials

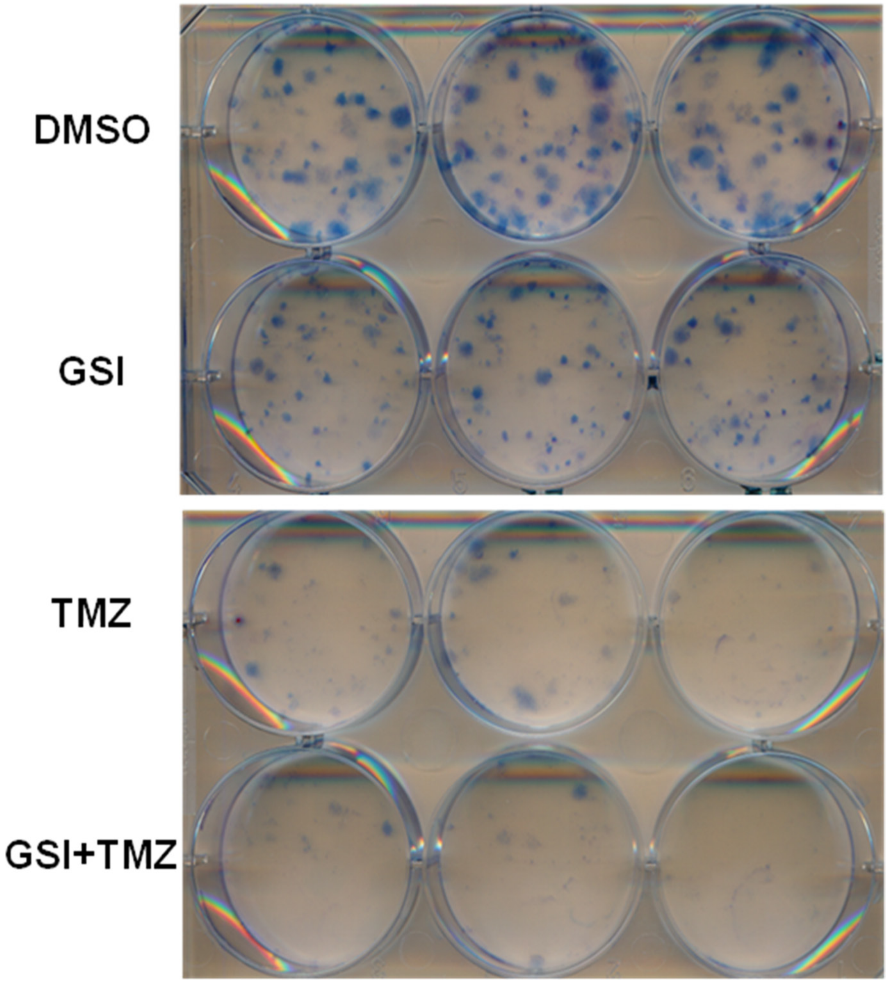

Supplementary Figure S1: E2 clonogenic assay. Representative image of the effect of GSI and TMZ treatment following radiation in E2 cells as measured by clonogenic assays.

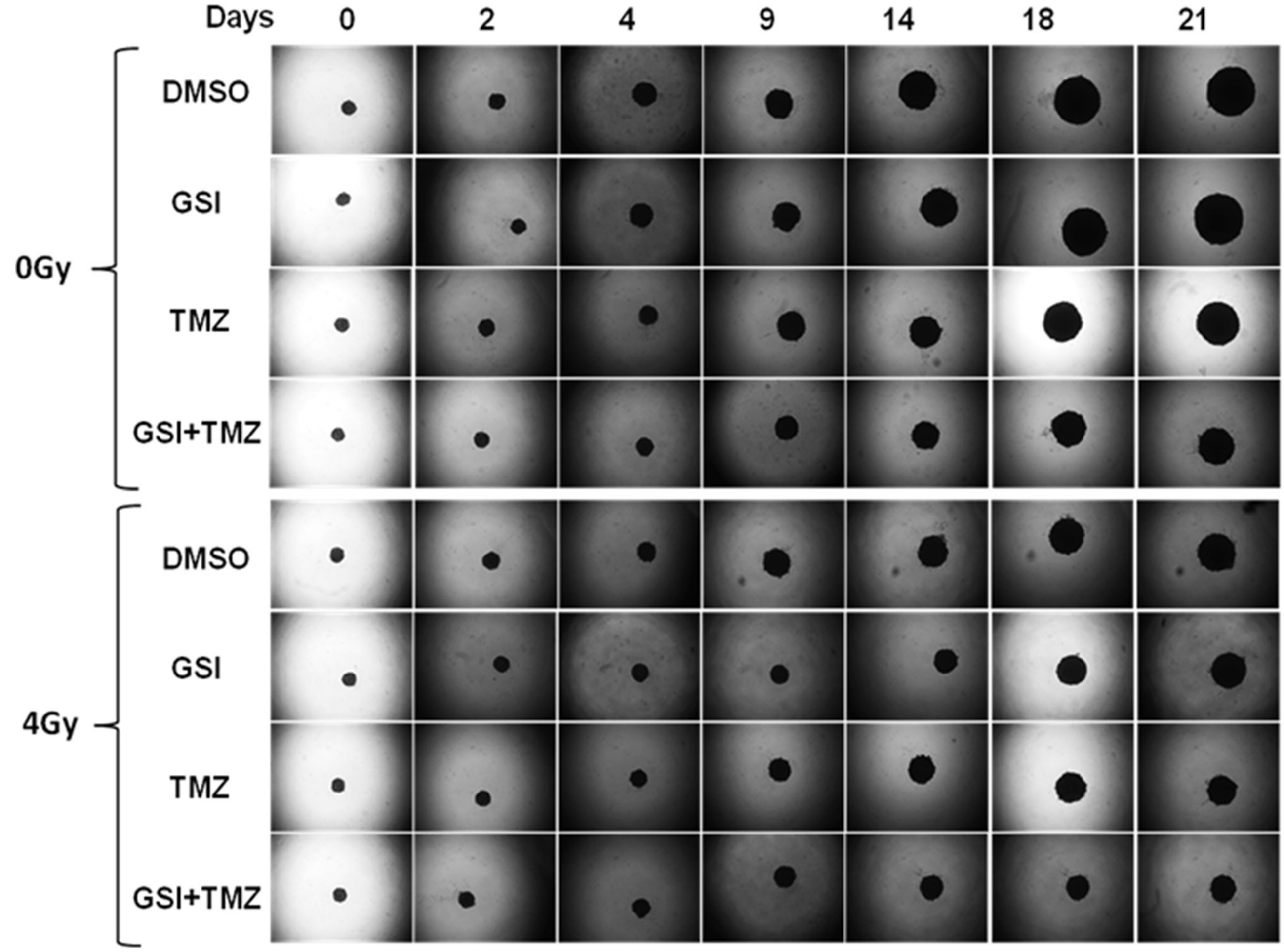

Supplementary Figure S2: U87MG-Luc2 spheroid assay. Representative image of the 3D spheroid upon indicated treatments over time.

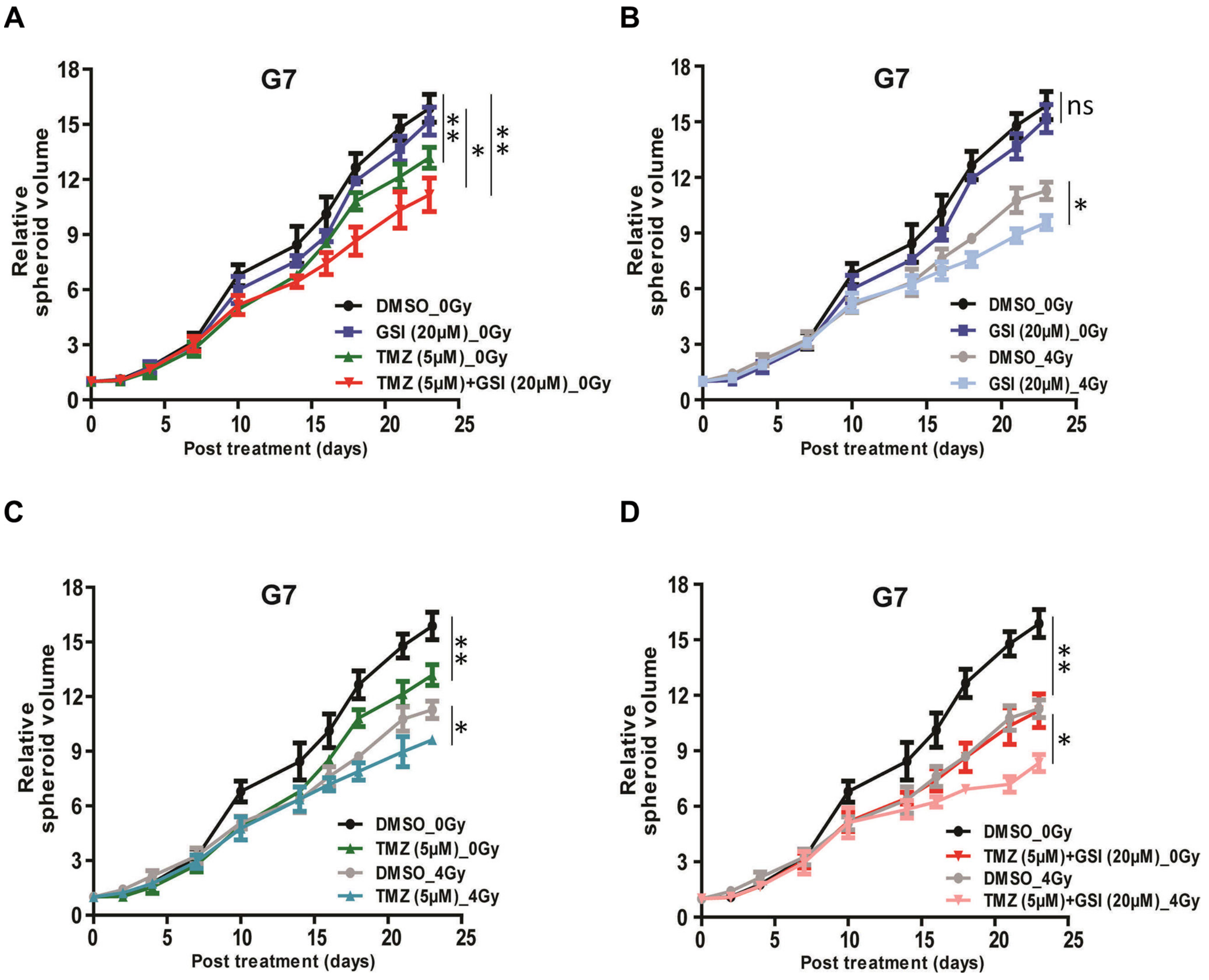

Supplementary Figure S3: Effect of NOTCH inhibition combined with TMZ and RT on 3D spheroid growth *in vitro*. (**A–D**) Individual spheroid growth in G7 cells was imaged 3 × /week and the volume was calculated after indicated treatments for 24 days post seeding. Spheroid growth demonstrates delays upon different treatment combinations compared with DMSO as vehicle control. Error bars indicates SEM. Asterisk indicates significance (*$p < 0.05$, **$p < 0.01$ and ***$p < 0.001$), ns: not significant.

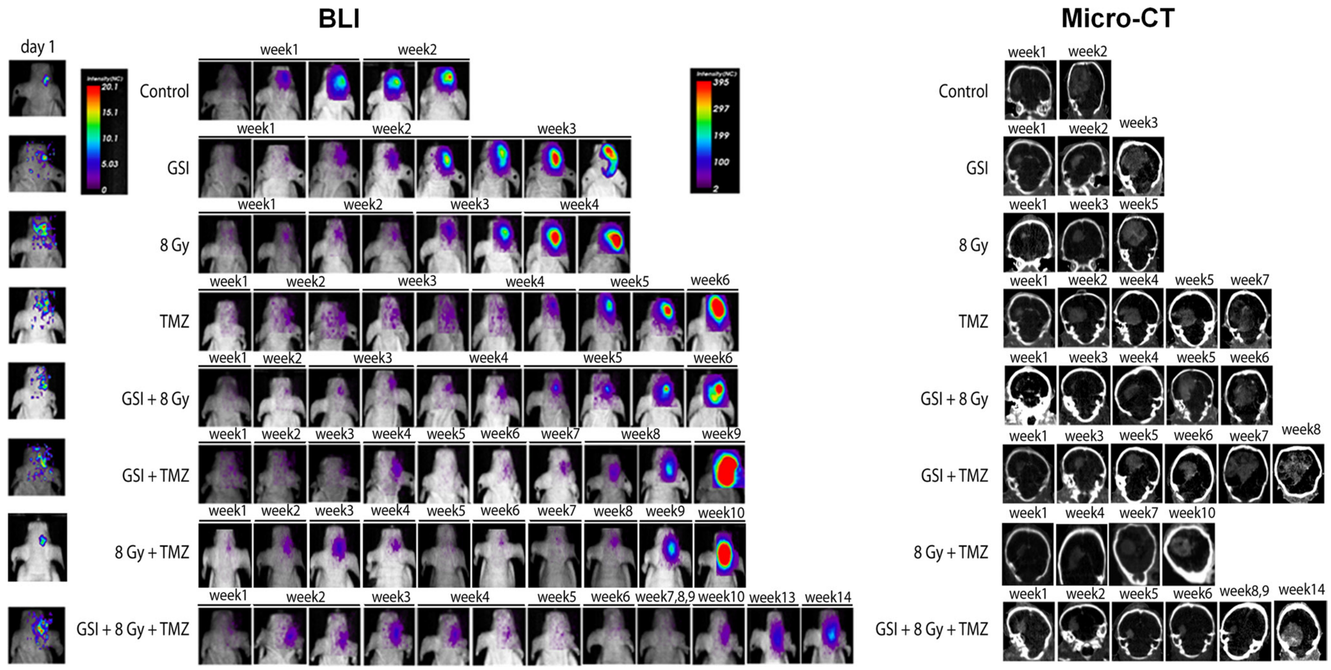

Supplementary Figure S4: Following tumor growth using BLI and/or micro-CT. Representative BLI and contrast enhanced micro-CT images of different treatment groups at different time points post-treatment.

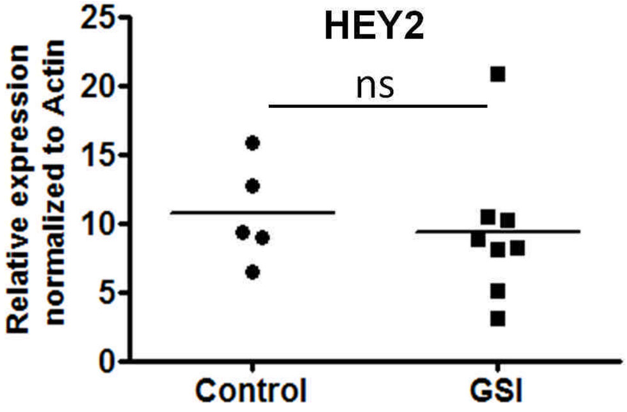

Supplementary Figure S5: NOTCH target gene expression upon treatment *in vivo*. mRNA expression of the HEY2 in tumor samples treated with GSI *vs.* control was determined by qRT-PCR. Values were normalized to Actin. (ns: not significant).

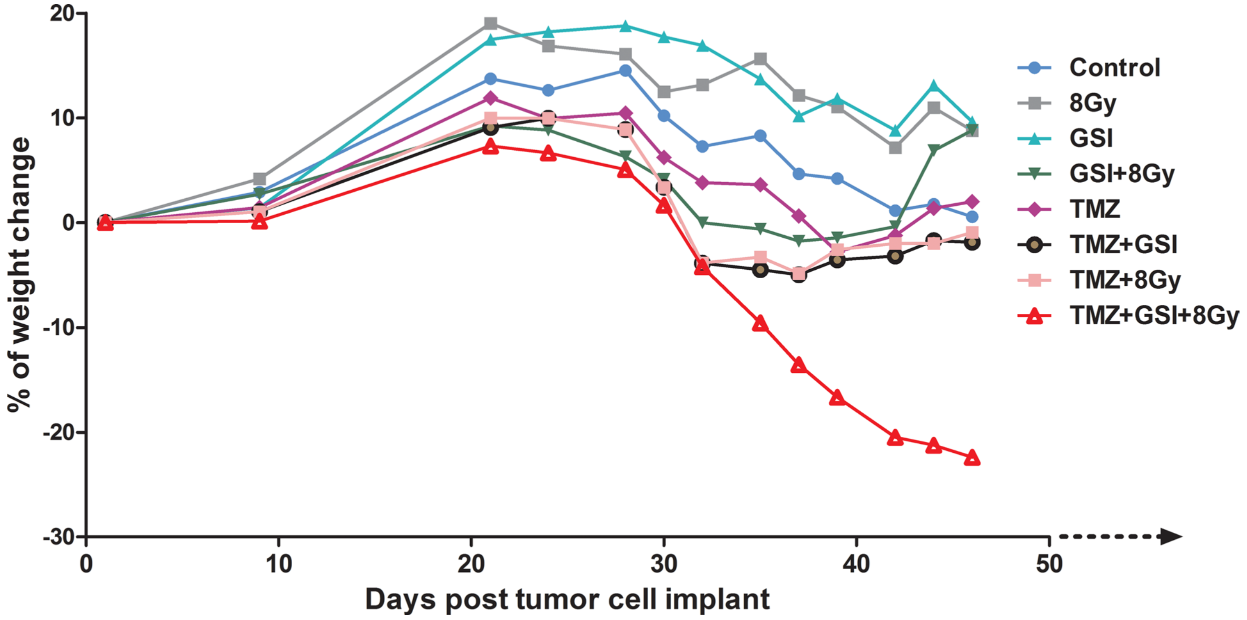

Supplementary Figure S6: Systemic toxicity (measured by % of change in body weight) of mice administered with different treatments as indicated. Day 0 is the start of the experiment after tumor cell implantation. After confirmation of the tumor establishment (between week 2–4), the treatment is started. The continues weight loss after week 3–4 in control, 8 Gy and GSI groups is due to the increase in the tumor size. Note reversible systemic toxicity in single treatment group (TMZ) as well as dual combination groups (GSI + 8 Gy, TMZ + GSI, TMZ + 8 Gy). 8 Gy and GSI alone did not enhance the systemic toxicity compared to the control. Triple combination resulted in significant dropout due to toxicity measured by > 20% weight loss in half of the mice in this group.
